# Potential for Bias in Prevalence Estimates when Not Accounting for Test Sensitivity and Specificity: A Systematic Review of COVID-19 Seroprevalence Studies

**DOI:** 10.3389/ijph.2025.1608343

**Published:** 2025-07-15

**Authors:** Sarah R. Haile, David Kronthaler

**Affiliations:** Epidemiology Department, Epidemiology, Biostatistics and Prevention Institute, University of Zurich, Switzerland

**Keywords:** prevalence, seroprevalence, diagnostic tests, statistical methods, Rogen-Gladen, bayesian, sensitivity, specificity

## Abstract

**Objectives:**

The COVID-19 pandemic has led to many studies of seroprevalence. A number of methods exist in the statistical literature to correctly estimate disease prevalence or seroprevalence in the presence of diagnostic test misclassification, but these methods seem to be not routinely used in the public health literature. We aimed to examine how widespread the problem is in recent publications, and to quantify the magnitude of bias introduced when correct methods are not used.

**Methods:**

A systematic review was performed to estimate how often public health researchers accounted for diagnostic test performance in estimates of seroprevalence.

**Results:**

Of the seroprevalence studies sampled, 77% (95% CI 72%–82%) failed to account for sensitivity and specificity. In high impact journals, 72% did not correct for test characteristics, and 34% did not report sensitivity or specificity. The most common type of correction was the Rogen-Gladen formula (57%, 45%–69%), followed by Bayesian approaches (32%, 21%–44%). Rates of correction increased slightly over time, but type of correction did not change.

**Conclusion:**

Researchers conducting studies of prevalence should report sensitivity and specificity of the diagnostic test and correctly account for these characteristics.

## Introduction

Since the beginning of the SARS-CoV-2 pandemic, thousands of papers have been published detailing seroprevalence estimates in various populations [1]. A glance into recent publications indicates that while some researchers used simple approaches such as proportions or logistic regression, others used complicated methods like Bayesian hierarchical models. An important question is therefore how often these methods are used in epidemiological studies and what, if any, degree of bias was introduced by using one method or the other.

As diagnostic tests are not 100% accurate, it is expected that some test results will be either false positives or false negatives. Using a simple proportion of the number of positive diagnostic tests over the total number of tests ignores any misclassification inherent to the test and may therefore be biased even when the study sample is representative of the study population. Many examples of this phenomenon are found in the literature [2, 3]. Statisticians often refer to sensitivity and specificity of the diagnostic test in relation to the degree of potential misclassification, but it is accepted that without a “gold standard” diagnostic tool, it is difficult to accurately assess disease prevalence.

Accounting for such misclassification in the interpretation of diagnostic tests is certainly not new in the literature. A straightforward method of adjusting observed prevalence, the Rogan-Gladen correction, is available [4, 5], which gives a maximum likelihood estimate of true prevalence assuming predefined test sensitivity and specificity and has been extended to compute confidence intervals [6, 7]. Recently, an adaptation of the Rogan-Gladen correction that accounts for sampling bias, for example, if only hospitalized subjects as opposed to the general population have been tested, has been proposed [8–10]. Bayesian approaches have also been developed [3, 11, 12]. A comparison of Bayesian and frequentist methods [13] showed that Bayesian methods, or the method of [4] with confidence intervals of [7] are to be preferred.

Despite this quite extensive treatment of the misclassification problem in the statistical literature, it appeared that many studies on COVID-19 seroprevalence were published without using any of the above mentioned methods. Many public health researchers appear to not realize they may be publishing biased results or do not know what to do about it. In order to assess how often such methods are used in practice, we performed a systematic review to estimate the proportion of recent publications estimating COVID-19 seroprevalence that do not correct for diagnostic test performance. We discuss these results for a real example of SARS-CoV-2 seroprevalence in children.

## Methods

### Calculation of Bias

Prevalence is the probability of having the disease of interest. Often in prevalence studies, this probability is studied at a specific point in time, giving so-called point prevalence [14]. Seroprevalence, a related concept, looks at the proportion of individuals in the population that have antibodies for a specific disease, for example, SARS-CoV-2 [15]. Given a diagnostic test’s sensitivity (denoted Se ), specificity (Sp) and true disease prevalence (P) , the bias when using the proportion of positive tests to estimate prevalence is (1−P)(1−Sp)−P(1−Se) . The Rogan-Gladen corrected estimate is $\frac{P_{\mathrm{obs}}+S_{p}-1}{Se+Sp-1}$ , where $P_{\mathrm{obs}}$ is the observed rate of positive tests. An overview of these terms and derivation of the bias estimate can be found in the Supplementary Methods, and Supplementary Table S1.

### Bounds on Bias

Suppose we want to guarantee that the bias is no larger than, say, δ=0.02 , that is ± 2% in either direction. We first note that the bias estimate given above is equivalent to 1−Sp+P(Sp+Se−2) and then solve

$$-\delta \leq 1-Sp+P\left(Sp+Se-2\right)\leq \delta$$

for P , to get:

$$\max\left(\frac{\delta +Sp-1}{Sp+Se-2},0\right)\leq P\leq \min\left(\frac{-\delta +Sp-1}{Sp+Se-2},1\right).$$

The lower bound will be 0 if δ≥1−Sp , while the upper bound will be 1 if δ≥1−Se . Therefore, if both Se and Sp are very high, say 99% or higher, the proportion of positive tests is a good estimate of the true prevalence. If only Se (or Sp ) is that high, this is will be true only when the true prevalence is quite high (low). When neither Se nor Sp is high, the proportion of positive tests may or may not be a good estimate of the true prevalence, depending on whether the missclassification errors approximately cancel each other out. See Supplementary Table S2 for a graphical representation of these bounds.

### Systematic Review

The systematic review of recent studies of seroprevalence in the literature started with a pubmed (https://pubmed.ncbi.nlm.nih.gov/) search for “COVID-19 seroprevalence”, which yielded 637 publications published in 2022. Publications were included in the systematic review if they assess COVID-19 seroprevalence in humans, and were published in 2022 in English or German. Exclusion criteria included: 1) studies solely comparing seroprevalence in different subgroups that do not report overall seroprevalence estimates, 2) studies solely examining risk factors for seropositivity that do not report overall seroprevalence estimates, 3) studies in animals, 4) reviews, 5) methodological papers, 6) studies with possible conflict of interest, or 7) if the full text was not available. During the screening process, it was additionally decided that research letters should be excluded in a sensitivity analysis, as it could often not be expected to include detailed information about the diagnostic test given word count restrictions. The following information was extracted: 1) whether the aim of the study was to assess COVID-19 seroprevalence in humans, 2) the sensitivity and 3) specificity of the diagnostic test, 4) the reported seroprevalence estimate (the first mentioned value, and if unadjusted was reported before adjusted, we extracted the most adjusted value of the first mentioned seroprevalence), and 5) which statistical methods were used to calculate seroprevalence. In some cases, publications noted the manufacturer of an assay, but not its sensitivity or specificity. If this information was not in the publication or its Supplementary Material, the study was counted as not having reported these characteristics. A protocol for the systematic review was developed using the PRISMA-P checklist (https://osf.io/b59x2). Two independent reviewers screened the publications using the rayyan.ai web-based tool, and performed data extraction in parallel using a structured spreadsheet. Discrepancies were resolved by discussion. It was later decided to add 1) stratification of results by journal quality and 2) examination of rates of adjustment over time. Therefore, we reexamined the publications to extract starting date of the sampling period. As journal impact factors vary greatly according to field and do not have a threshold above which a journal is considered “high quality”, classification of journals proceeded according to the quartile of the SCImago Journal Rank [16]. Journal rankings for 2024 (or the most recent available year) were taken from the Scimago website (www.scimagojr.com) [17], and categorized as Q1 (“high impact”) or Q2-Q4 (see Supplementary Material).

Summary statistics were computed for the methods used (n (%)), reported sensitivity and specificity (median [range]) and estimated bias (median [range]). The analysis was also stratified by type of journal (high impact vs. other), and changes over time were examined through stratification by year and quarter of the beginning of the sampling period. We further categorized the bias into categories for analysis at the following thresholds: −15%, −10%, −5%, −1%, 1%, 5%, 10%.

### COVID-19 Example

To provide a concrete example of this problem, we use the Ciao Corona study [18], a school-based longitudinal study of seroprevalence in Swiss school children with 5 rounds of SARS-CoV-2 antibody testing between June 2020 and June 2022, covering a range of seroprevalences in the population (Trial Registration: ClinicalTrials.gov NCT04448717). The study was conducted in accordance with the Declaration of Helsinki and approved by the Ethics Committee of the Canton of Zurich, Switzerland (2020-01336). All participants provided written informed consent before being enrolled in the study.

### Patient and Public Involvement

It was not appropriate or possible to involve patients or the public in the design, or conduct, or reporting, or dissemination plans of our research.

## Results

### Systematic Review

**FIGURE 1:**
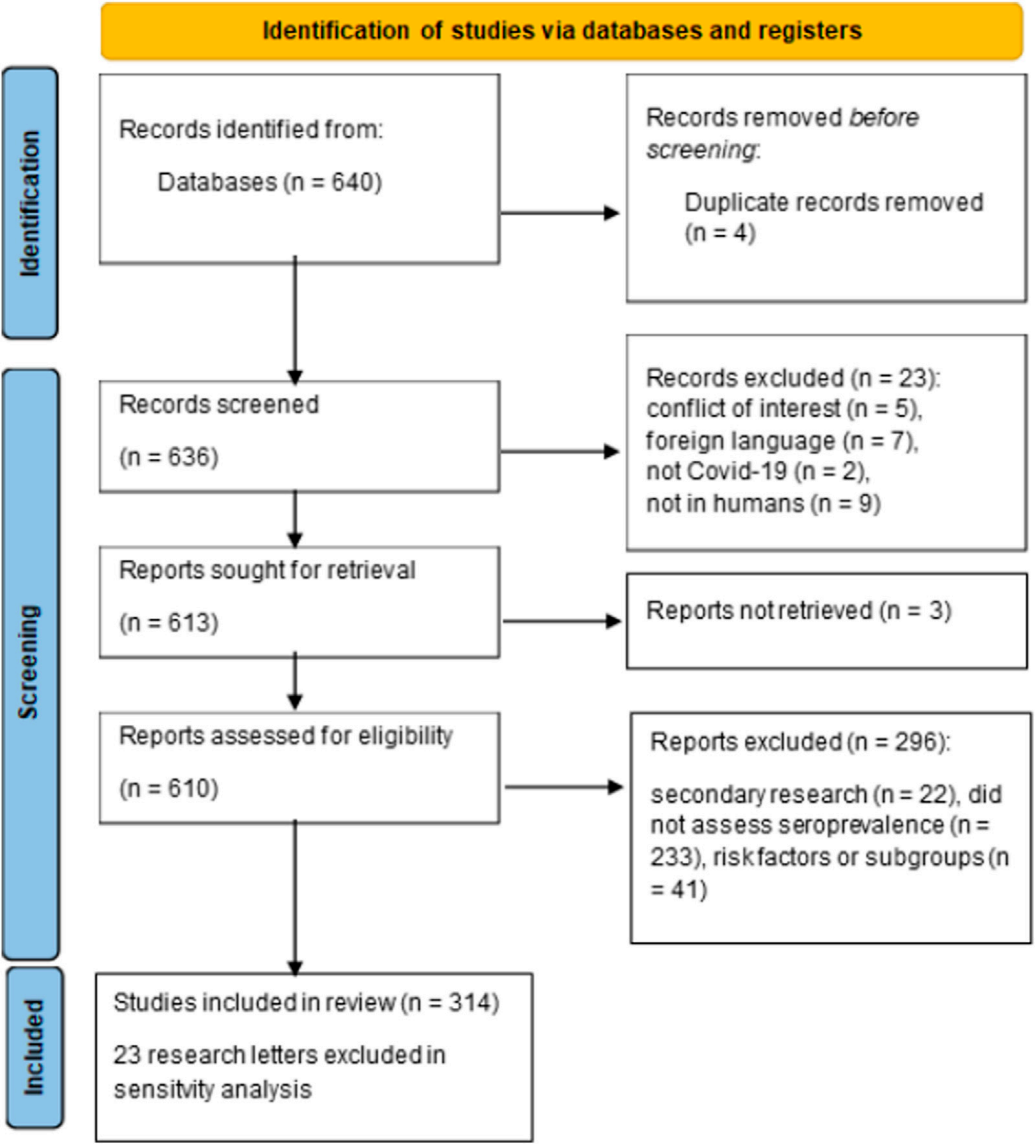
PRISMA diagram for systematic review (Zurich, Switzerland. 2023).

To examine the methods actually used in seroprevalence studies in the literature, we performed a systematic review of publications from 2022 which estimated COVID-19 seroprevalence in humans (Table 1). Of the 640 publications identified 314 were included in the systematic review (Figure 1; Supplementary Table S1). Of the included publications, 76% (n = 240, 95% CI 71.3%–81.0%) did not adjust for diagnostic test performance, while 22.6% corrected for sensitivity and specificity of the diagnostic test (n = 71, 18.1%–27.6%). Among the publications which adjusted for test characteristics, 41 (13.0%) used the Rogan-Gladen correction, 23 (7.3%) used Bayesian approaches, and 8 (2.5%) mentioned adjustment but did not specify the method used. High impact journals were more likely to report corrected seroprevalence estimates (27.8%, 60/216 vs. 12.2%, 12/98), but even in this subset of journals 72% of publications did not correct for properties of the diagnostic test (Table 1). All instances of Bayesian approaches to account for diagnostic accuracy however came from high impact journals.

**TABLE 1:** Key outcomes of systematic review [n (%), median (min - max)]. The main analysis included 314 publications meeting all inclusion criteria. High impact journals included are listed in the Supplementary Material. (Zurich, Switzerland. 2023).

Characteristic	Overall (N = 314)	High impact (N = 216)	Other journals (N = 98)
Start of sampling period			
Median	2020-09-24	2020-10-15	2020-09-15
(Min - Max)	(2019-10-15 - 2022-07-01)	(2019-10-15 - 2022-04-26)	(2019-11-15 - 2022-07-01)
Correction/reported Se + Sp			
Corrected and fully reported Se/Sp	67 (21%)	56 (26%)	11 (11%)
Corrected and no/partial information	5 (1.6%)	4 (1.9%)	1 (1.0%)
Uncorrected and fully reported Se/Sp	122 (39%)	82 (38%)	40 (41%)
Uncorrected and no/partial information	120 (38%)	74 (34%)	46 (47%)
Statistical method			
Bayesian	23 (32%)	23 (38%)	0 (0%)
Rogan-Gladen	41 (57%)	33 (55%)	8 (67%)
Unspecified method	8 (11%)	4 (6.7%)	4 (33%)
Sensitivity	95.1 (60.2 - 100.0)	95.0 (60.2 - 100.0)	96.8 (72.2 - 100.0)
Specificity	99.6 (82.4 - 100.0)	99.6 (82.4 - 100.0)	99.8 (92.5 - 100.0)
Expected bias	−0.01 (−12.20 - 9.10)	−0.03 (−12.20 - 9.10)	0.09 (−6.30 - 7.15)
expected bias (category)			
[-15,-10)	5 (2.6%)	5 (3.6%)	0 (0%)
[-10,-5)	8 (4.2%)	6 (4.3%)	2 (3.9%)
[-5,-1)	29 (15%)	20 (14%)	9 (18%)
[-1,1)	116 (61%)	84 (61%)	32 (63%)
[1,5)	28 (15%)	22 (16%)	6 (12%)
[5,10]	3 (1.6%)	1 (0.7%)	2 (3.9%)

### Reporting of Sensitivity and Specificity

Further, among those publications that did not adjust for test performance, 122/242 (50.4%) reported sensitivity and specificity, while the remaining publications either did not report test characteristics (45.0%, n = 109) or only reported partial test characteristics (4.5%, n = 11). Among all publications reviewed, it is therefore observed that 34.7% (109/314) neither adjusted for test performance nor reported sensitivity and specificity. Among those that did not correct for test performance but did report both sensitivity and specificity (n = 122), expected bias ranged from −12.2% to 7.1%. 73 (60%) of the publications reporting seroprevalence to within ± 1% of the true value despite not using any adjustment, while the remaining 49 (39.5%) needed adjustment for test performance (9 of those were not even within ± 5%). It could be inferred therefore that approximately 48 of the 120 publications not or partially reporting test performance are also in need of adjusted seroprevalence estimates to account for test performance, even though all of those publications reported naive estimates. Even among high impact journals, only 28% (60/216) of publications corrected for test characteristics (compared to 11% among lower quality journals) and 34% (74/216) of publications neither corrected for test performance nor provided sensitivity and specificity (47%) in other journals.

### Association With Sampling Period

**FIGURE 2:**
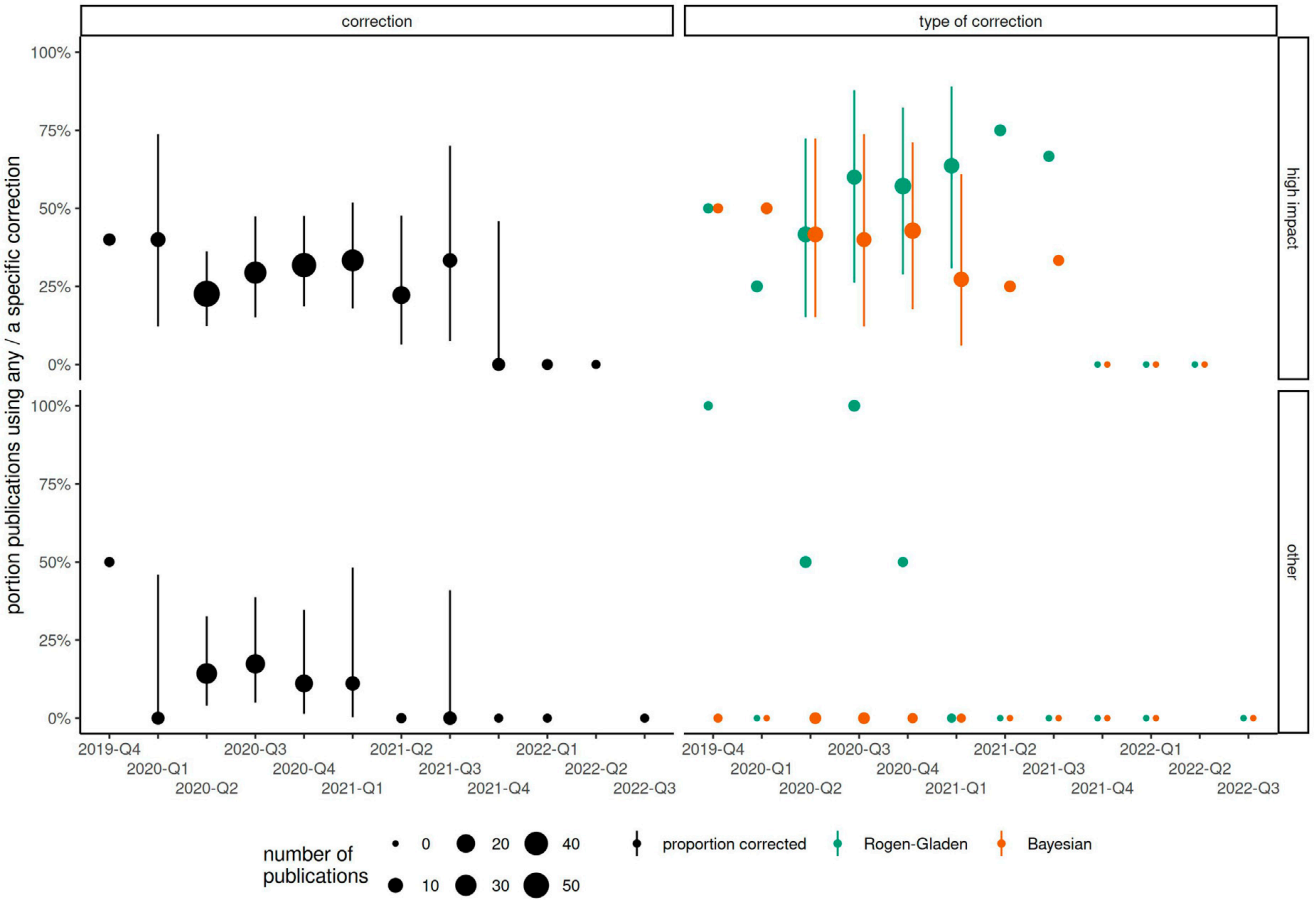
Changes in proportion of publications correcting for test sensitivity and specificity, by start of sampling period (categorized by year-quarter) and type of journal. Point size corresponds to the total number of publications. (Zurich, Switzerland. 2023).

**FIGURE 3:**
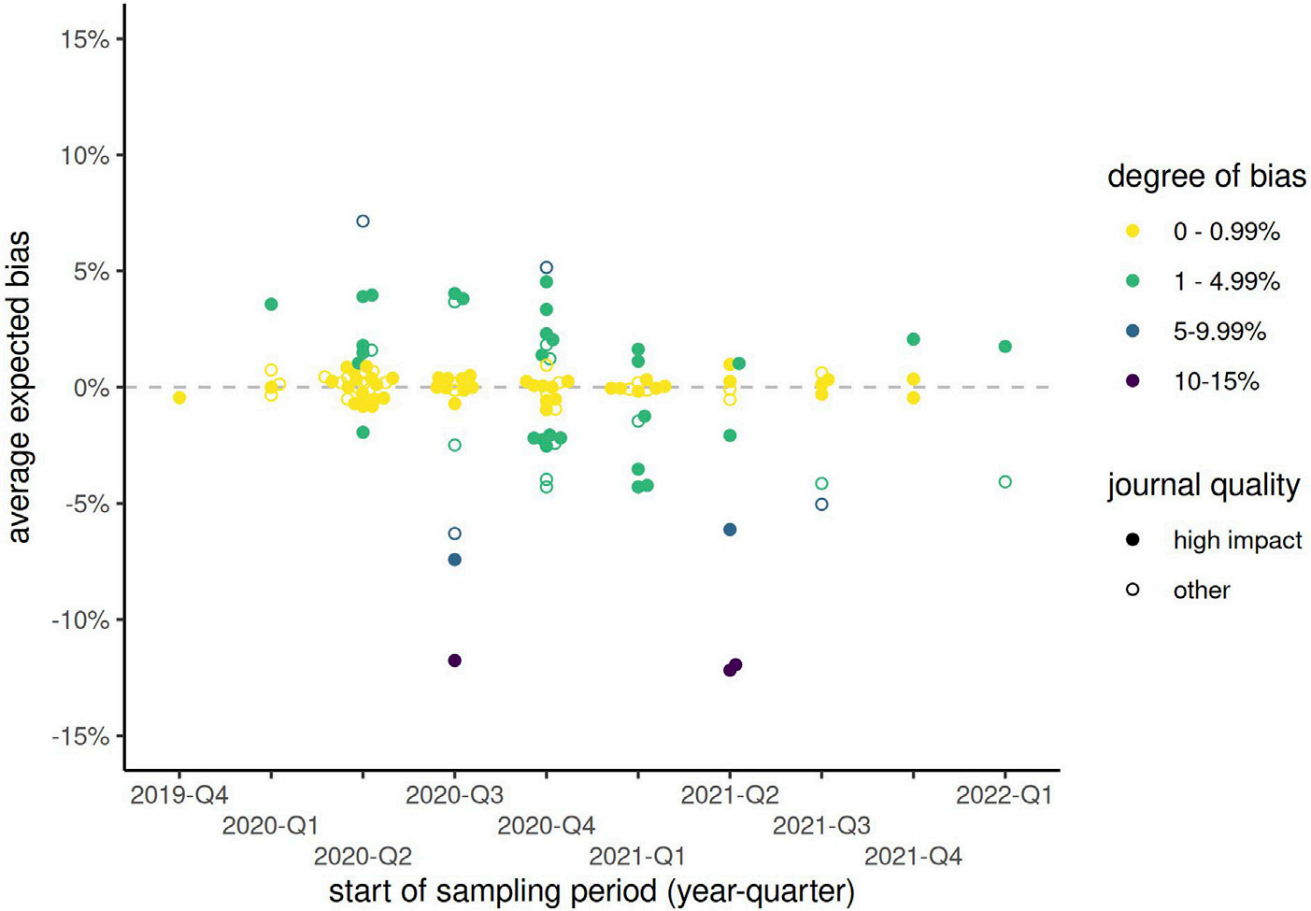
Expected bias by start of sampling period (categorized by year-quarter) and type of journal, among all publications reporting both sensitivity and specificity but not correcting for these test characteristics. Solid points represent publications from high impact journals, while hollow points show those from other journals. (Zurich, Switzerland. 2023).

The sampling period of included publications started as early as October 2019 (retrospectively, using, e.g., blood samples), or as late as July 2022, with interquartile range between May 2020 and January 2021. Among the 53 publications from high impact journals with sampling period in the 2nd quarter of 2020, 23% (n = 12) corrected for test sensitivity and specificity using any method. The proportion of corrected results had increased slightly to 11/33 (33%) by the 1st quarter of 2021 (Figure 2; Supplementary Table S2). Therefore, even 1 year into the COVID-19 pandemic, 2/3 of publications in high impact journals did not correct for test characteristics. In papers published in lower impact journals, rates of correction remained below 20% at all timepoints and appeared to vary little over time. At most time points, Rogan-Gladen corrections were more common than those using more complex Bayesian methods (e.g., in 2021 Q2, 64% Rogan-Gladen vs. 27% Bayesian among 11 papers in high impact journals). Expected bias increased over time, with increasingly more publications having an absolute expected bias above 1% (Figure 3; Supplementary Table S3), while the average expected bias tended to be negative in later time periods. For example, in the 2nd quarter of 2020, 8 of 34 (24%) publications that had not corrected for test characteristics but had reported both sensitivity and specificity had an expected average bias above 1%, compared with 16/28 (57%) in the 4th quarter of 2020, or 7/16 (44%) in the 1st quarter of 2021.

### Sensitivity Analysis

These results did not change when excluding publications denoted “research letters” or similar (Supplementary Table S4). Among 99 publications from high impact journals, 34% (n = 36) corrected for sensitivity and specificity, and a further 43% at least reported these quantities, compared to 16% (n = 34 of 209) correcting and 43% reporting sensitivity and specificity in other journals. Nevertheless, 23% of publications from top journals neither corrected nor reported sensitivity and specificity, compared to 41% of publications from other journals.

### Example

As an example of what happens when test sensitivity and specificity are not accounted for in the statistical analysis, take the Ciao Corona study [18], a school-based longitudinal study of seroprevalence in Swiss school children with 5 rounds of SARS-CoV-2 antibody testing between June 2020 and June 2022. The antibody test used has a sensitivity of 94% in children, and a specificity of 99.2%. In June 2020, 98/2473 (4.0%) of subjects showed as seropositive, compared to 154/2500 (6.2%) in October 2021, 17.3% (426/2453) in March 2021, 48.5% (910/1876) in November 2021, and 94.5% (2008/2125) in June 2022. Given the diagnostic test characteristics, absolute bias can be expected to be less than 1% in the range of 0%–26.5% disease prevalence, and less than 2% for disease prevalence of up to 41.2%. These results imply that reported seroprevalence estimates based on a naive logistic approach are likely relatively unbiased for the first 3 rounds of Ciao Corona antibody testing (0.5%, 0.4% and −0.4% respectively), but that after that any seroprevalence estimates that do not adjust for test characteristics are likely quite biased (−2.4% and −5.6%). In order to adjust for covariates and survey sampling weights, we corrected the seroprevalence estimates using a Bayesian hierarchical model approach in all rounds of testing.

## Discussion

We have demonstrated that many public health researchers are not aware of methods for reducing bias in seroprevalence estimates, as even in high impact journals less than 30% of publications corrected for test characteristics, and 34% did not even report this information. The rate of correction only improved slightly during the first year of the COVID-19 pandemic, and the type of correction changed little over time. Importantly, if uncorrected seroprevalence estimates continued to be published without reporting test sensitivity or specificity even in the third year of a global pandemic in high impact journals, it would appear that many peer reviewers and journal editors also fail to notice this widespread problem. While the average expected bias over all studies is close to 0, some studies had expected absolute bias of 5%, 10% or even higher. Inclusion of such studies in a systematic review of seroprevalence, for example, could be extremely problematic, and further bias such meta-analyses. Moreover, biased seroprevalence estimates may influence scientific discourse and inform policy decision, particularly given that many of the studies in our review are published in high-impact journals or are frequently cited.

These results emphasize the necessity in public health research to not simply report raw proportions of positive tests, even if those are adjusted for demographic characteristics using, e.g., logistic regression. Since disease prevalence is of course not known precisely prior to study conduct, the most straightforward approach is then to plan statistical methods so that sensitivity and specificity are accounted for. Even if other sources of bias (e.g., sampling bias, or sampling variation) are accounted for, the results of seroprevalence studies will continue to be biased if analyses do not also account for test sensitivity and specificity. Care should also be taken in reading publications reporting (sero)prevalence estimates to ensure that suitable statistical methods have been used.

We have not adjusted for demographic characteristics, such as age and gender, or used weighting to approximate the target population, as is typical in surveys of disease prevalence. However, such adjustment cannot alleviate any general concerns of bias as presented here. The average bias reported does not account for other possible issues with a diagnostic test [9, 19–21], that can often not be corrected with statistical methods (e.g., when the validation sample, on which the sensitivity and specificity estimates are based, is not similar to the population of interest). In some cases, the name and/or manufacturer of the diagnostic test was reported but sensitivity and specificity were not. It is therefore possible that some missing sensitivity and specificity details could have been extracted, but it is not clear that what can be found, e.g., on the manufacturer’s website currently is the same as it was when the testing took place.

The question remains as to how best to account for diagnostic test sensitivity and specificity when estimating disease prevalence. A nice outline of some appropriate methods along with implementation in R [22] code is given by [13, 23, 24]. To calculate corrected confidence intervals for prevalence in studies where covariates do not need to be adjusted for, and no survey weights are needed, the R package bootComb [25] and website “epitools” (https://epitools.ausvet.com.au/trueprevalence) are available, while Bayesian methods are available in prevalence [26]. Using the Rogan-Gladen correction with bootstrap confidence intervals, or the Bayesian correction in the prevalence package are appropriate when there is no need to adjust for any other factors. Adjusting for covariates, adjusting for sampling bias or variation, or application of post-stratification weights (among other issues) may unfortunately need to be done without the use of such prepackaged code, e.g., as described by [27]. Collaboration with experienced statisticians is invaluable in ensuring that correct analysis techniques are used so that unbiased prevalence estimates can be reported.

The majority of publications, even in high impact journals, reporting seroprevalence estimates in the literature do not account for sensitivity and specificity of the diagnostic test, even though the need to adjust seroprevalence estimates for test performance is well known the statistical literature. Public health researchers performing prevalence studies should consult experienced statisticians when analyzing such data, and be sure to account for test performance. However, researchers reviewing published prevalence studies also need to be aware of this issue. Perhaps reporting guidelines for prevalence studies, such as CONSORT for observational studies [28], are necessary. The results here will assist reviewers in determining the magnitude of bias that can be expected, so that publications in the epidemiology literature can be interpreted properly.
