# Supplementary material for: Potential for Bias in Prevalence Estimates when Not Accounting for Test Sensitivity and Specificity: A Systematic Review of COVID-19 Seroprevalence Studies
Source: Int J Public Health. 2025 Jul 15;70:1608343. doi: 10.3389/ijph.2025.1608343 (PMC12303856; doi:10.3389/ijph.2025.1608343)
Supplement: Supplementary file 1 [file DataSheet2.pdf]

**Potential for bias in prevalence estimates when not accounting for test sensitivity and specificity: a systematic review of COVID-19 seroprevalence studies - Supplementary Material**

# Supplementary Methods

Here we provide a short overview of the concepts of sensitivity and specificity. Further information can be found in e.g. (1–3). First, we introduce some notation. Disease status, $D$, is denoted 1 if a subject has the disease in question (or for the case of seroprevalence, has antibodies for it), and 0 otherwise. Similarly, the result of the diagnostic test, $Y$, is given as 1 if the subject tests positive for the disease, and 0 otherwise. FP is often used to refer to false positive test results, and similarly FN for false negatives, TN for true negatives and TP for true positives.

Sensitivity, denoted $Se$, sometimes also called the true positive fraction (TPF), is the probability of having a positive test result, given that the subject has the disease, $Pr(Y = 1|D = 1)$ (1). On the other hand, specificity, $Sp$ is the probability of having a negative test result when a subject does not have the disease, $Pr(Y = 0|D = 0)$ (sometimes 1 - specificity is discussed, which is often referred to as false positive fraction, or FPF (2)). In real settings where true disease status is known via another method, sometimes referred to as the "gold standard", $Se$ can be computed as $TP/(TP + FN)$, where TP is the number of true positives and FN is the number of true negatives. Similarly, $Sp$ can be computed as $1 - FP/(FP + TN)$.

The proportion of positive tests can be expressed as

$$Pr(Y = 1) = (FP + TP)/(FP + TP + TN + FN),$$

while the disease prevalence in the sample can be expressed as

$$Pr(D = 1) = (FN + TP)/(FP + TP + TN + FN).$$

The difference between these two quantities is simply $(FP - FN)/(FP + TP + TN + FN)$, that is, the proportion of false positives minus the proportion of false negatives.

According to the definition of joint probability $Pr(A, B) = Pr(A|B)Pr(B)$, the proportion of false positives can be written as

$$Pr(Y = 1, D = 0) = Pr(Y = 1|D = 0)Pr(D = 0),$$

which simplifies to $(1 - P)(1 - Sp)$. In a similar fashion, the proportion of false negatives can be written as

$$Pr(Y = 0, D = 1) = Pr(Y = 0|D = 1)Pr(D = 1),$$

which simplifies to $P(1 - Se)$. The bias when using the proportion of positive tests, $Pr(Y = 1)$, to estimate the proportion with disease, $Pr(D = 1)$, is therefore

$$(1 - P)(1 - Sp) - P(1 - Se)$$

or equivalently

$$1 - Sp + P(Sp + Se - 2).$$

One simple way to correct for this bias, if no dependence on covariates is assumed, is to use the approach of Rogan and Gladen (4) which gives the corrected prevalence as

$$P_{RG} = \frac{P_{obs} + Sp - 1}{Se + Sp - 1},$$

assuming an observed fraction $P_{obs}$ of positive test results. In a small number of cases, primarily when the sample size and the prevalence are both small (5,6), the Rogan-Gladen correction will yield values less than 0 or greater then 1. However, even if this “clipped” version has some bias, the variance will be smaller.

**Expected bias** Next, we sought to characterize scenarios where expected bias would be

minimal. Using the result $\text{bias} = 1 - Sp + P(Sp + Se - 2)$ described above, we calculated the expected bias for a range of reasonable combinations of sensitivity, specificity and disease prevalence (Figure S1). When sensitivity and specificity were both 90%, bias was as high as 10%, especially near prevalences of 0% or 100% (solid line in leftmost panel of Figure S1). When specificity was 90%, a bias of 10% could be expected with small prevalences near 0% even if sensitivity was 99%. The least bias, 1%, could be expected where sensitivity and specificity were both 99%.

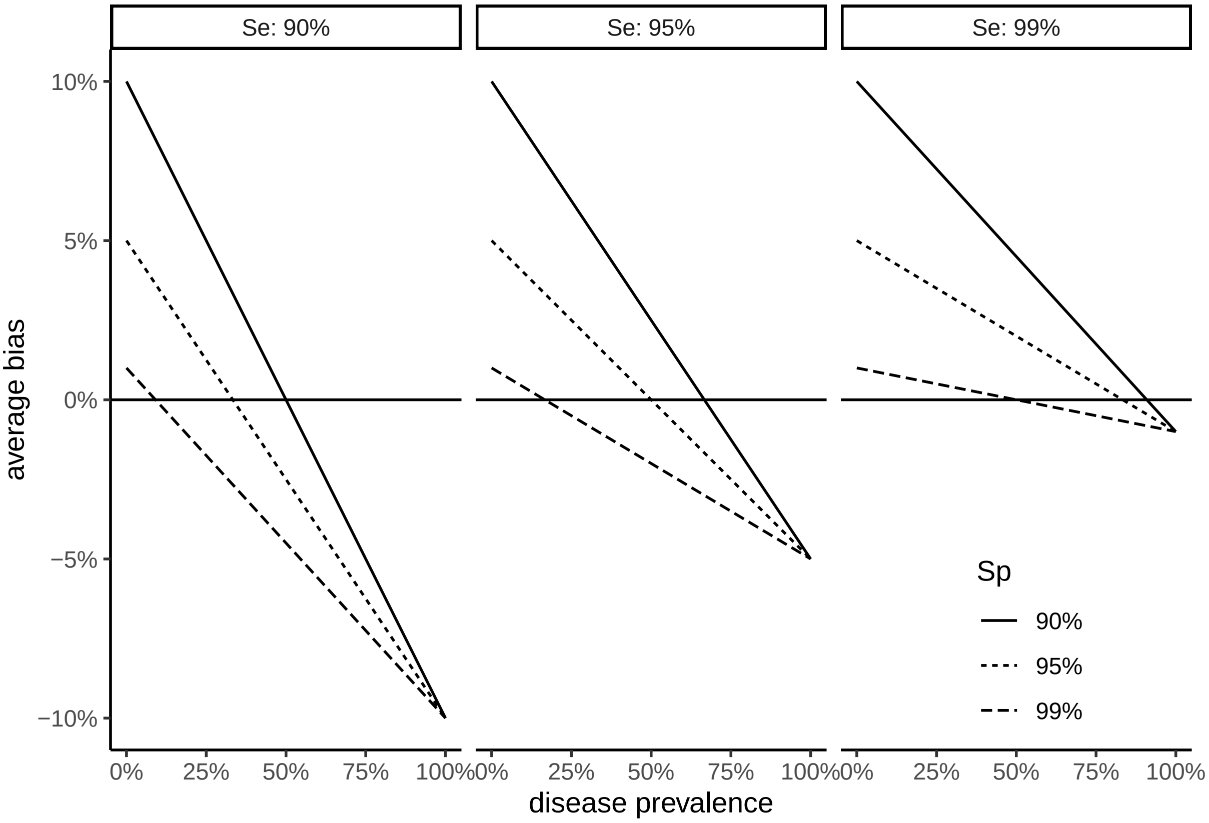


Figure S1: Epected bias in prevalence estimate for selected combinations of sensitivity, specificity and true disease prevalence

**Minimum tolerated bias** We explored where the maximum tolerated bias is limited to 1%, 2.5% and 5% (Figure S2). When $Se$ and $Sp$ are each 90%, bias is within a tolerance of 1% only very close to 50% disease prevalence, within 2.5% tolerance in the range of 38% - 62% disease prevalence and to within 5% tolerance as long as disease prevalence is between 25% and 75%. When the desired tolerance is 1%, the range of disease prevalence where a naive approach will yield unbiased results is fairly narrow in all cases, unless $Se$ and $Sp$ are each at least 99%. Outside of these ranges, using the proportion of positive test results

to estimate seroprevalence will be considerably biased, and more sophisticated analysis methods should be used.

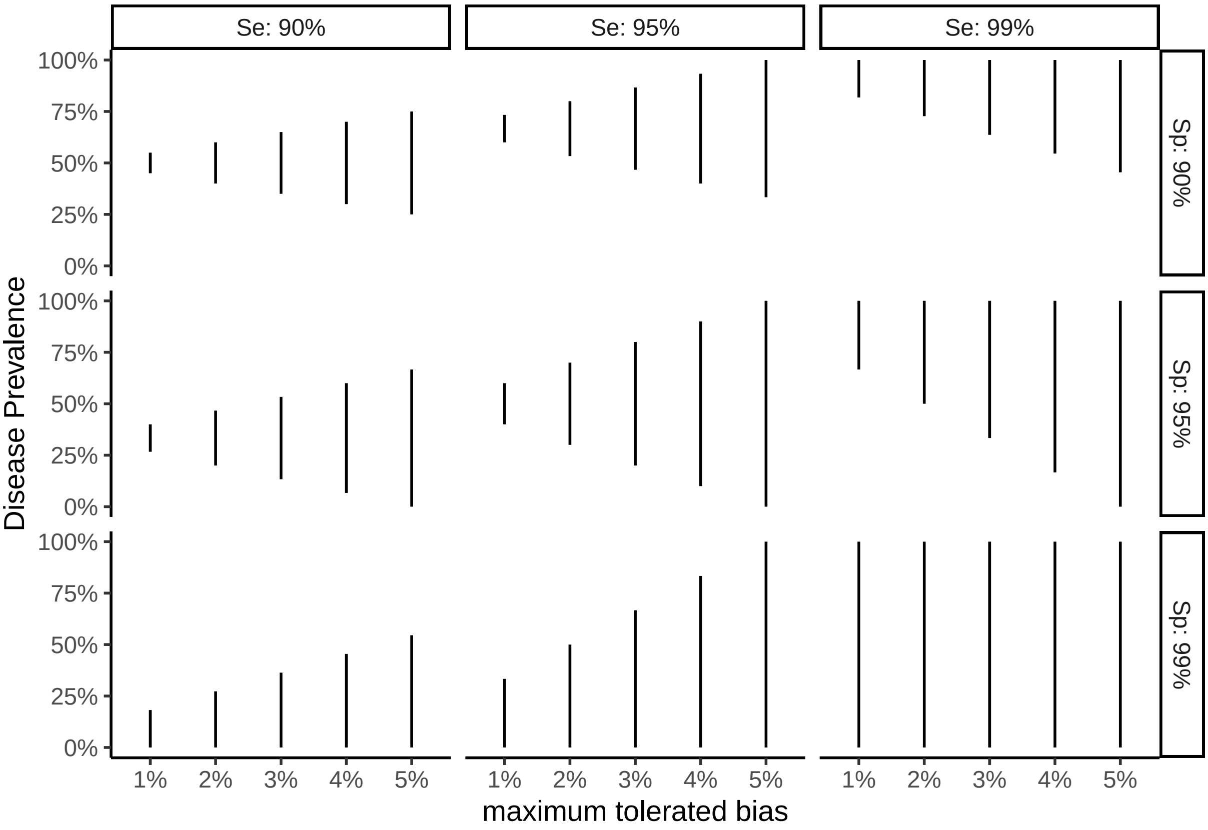


Figure S2: Range of true disease prevalence where the rate of positive tests is a close approximation of disease prevalence.

1. Altman D, Bland J. Statistics notes: Diagnostic tests 1: Sensitivity and specificity. BMJ. 1994;308(1552).

2. Pepe M. The statistical evaluation of medical tests for classification and prediction. OUP; 2003.

3. Lewis F, Torgerson P. A tutorial in estimating the prevalence of disease in humans and animals in the absence of a gold standard diagnostic. Emerging Themes in Epidemiology. 2012;9(9).

4. Rogan W, Gladen B. Estimating prevalence from the results of a screening test. Am J Epidemiol. 1978;107(41).

5. Hilden J. A further comment on "estimating prevalence from the results of a screening test'. Am J Epidemiol. 1979;109(6):721–3.

6. Kritsotakis E. On the importance of population-based serological surveys of SARS-CoV-2 without overlooking their inherent uncertainties. Public Health in Practice. 2020;1:10013.

# Journals in systematic review, by SCImago classification

## Q1 journals of publications included in the systematic review

Am J Emerg Med, Am J Public Health, BMC Infect Dis, BMC Public Health, BMJ Open, CMAJ, CMAJ Open, Can J Public Health, Clin Infect Dis, Clin Microbiol Infect, Elife, Emerg Infect Dis, Emerg Microbes Infect, Environ Health Prev Med, Epidemics, Epidemiol Serv Saude, Eur J Clin Microbiol Infect Dis, Eur J Gen Pract, Eur J Immunol, Eur J Pediatr, Euro Surveill, Fam Pract, Front Cell Infect Microbiol, Front Public Health, HIV Med, Health Secur, Infect Control Hosp Epidemiol, Infect Dis (Lond), Infection, Influenza Other Respir Viruses, Int J Epidemiol, Int J Gynaecol Obstet, Int J Infect Dis, Int J Public Health, J Acquir Immune Defic Syndr, J Adolesc Health, J Am Geriatr Soc, J Cancer Res Clin Oncol, J Clin Epidemiol, J Clin Virol, J Community Health, J Cyst Fibros, J Epidemiol, J Glob Health, J Infect, J Infect Dis, J Infect Public Health, J Int Assoc Provid AIDS Care, J Med Virol, J Neurol, J Pediatric Infect Dis Soc, J Public Health (Oxf), J Racial Ethn Health Disparities, JAMA, JAMA Netw Open, MMWR Morb Mortal Wkly Rep, Med J Aust, Med Microbiol Immunol, Microbiol Spectr, Mult Scler, N Engl J Med, Nat Commun, Occup Environ Med, PLoS One, Paediatr Perinat Epidemiol, Pediatr Blood Cancer, Pediatr Infect Dis J, Public Health, Public Health Rep, Respir Res, Rheumatology (Oxford), Rural Remote Health, Scand J Public Health, Sci Rep, Sex Transm Infect, Transbound Emerg Dis, Trop Med Int Health, Viruses, World J Pediatr, mSphere

## Q2-Q4 journals of publications included in the systematic review

Acta Clin Belg, Acta Med Port, Acta Odontol Scand, Acta Parasitol, Acta Virol, Am J Med Sci, Am J Trop Med Hyg, Ann Saudi Med, Ann Work Expo Health, Arch Virol, Biomed Res Int, Blood Transfus, Braz J Otorhinolaryngol, Braz Oral Res, Bull Exp Biol Med, Cancer Invest, Clin Exp Rheumatol, Clin Rheumatol, Clinics (Sao Paulo), Dan Med J, Dtsch Arztebl Int, Enferm Infecc Microbiol Clin (Engl Ed), Epidemiol Health, Epidemiol Infect, Eur Rev Med Pharmacol Sci, Hong Kong Med J, Immun Inflamm Dis, Indian J Med Microbiol, Indian J Med Res, Indian J Public Health, Infect Dis Health, Infect Dis Now, Int J Environ Res Public Health, Int J Immunopathol Pharmacol, Int J Occup Med Environ Health, Int J STD AIDS, Ir J Med Sci, Isr Med Assoc J, J Emerg Nurs, J Epidemiol Glob Health, J Infect Dev Ctries, J Korean Med Sci, J Med Life, J Med Microbiol, J Nippon Med Sch, J Occup Environ Med, JPEN J Parenter Enteral Nutr, Joint Bone Spine, Jpn J Infect Dis, Med Klin Intensivmed Notfmed, Med Pr, Medwave, Natl Med J India, Occup Med (Lond), Oncol Res Treat, Ophthalmic Epidemiol, Oral Health Prev Dent, Pathog Glob Health, Rev Inst Med Trop Sao Paulo, Rev Soc Bras Med Trop, Salud Publica Mex, Swiss Med Wkly, Trans R Soc Trop Med Hyg, Transfus Apher Sci, Transfus Clin Biol, Vector Borne Zoonotic Dis

## Q1 journals of publications excluded from the systematic review

ACS Biomater Sci Eng, Acta Paediatr, Acta Trop, Am J Epidemiol, Am J Infect Control, Ann Emerg Med, Ann Epidemiol, Ann Hepatol, Ann Intern Med, Antimicrob Resist Infect Control, Appl Microbiol Biotechnol, Arthritis Care Res (Hoboken), BMC Med, BMJ Glob Health, Biochem Med (Zagreb), Biomed Pharmacother, Biosensors (Basel), Br J Haematol, Bull Math Biol, Clin Chem Lab Med, Clin Exp Immunol, Cochrane Database Syst Rev, Endocr Pract, Environ Res, Epidemiology, Eur Arch Psychiatry Clin Neurosci, Eur J Cancer, Eur J Epidemiol, Front Immunol, Front Med, Health Policy Plan, Health Rep, J Clin Immunol, J Clin Microbiol, J Clin Nurs, J Crohns Colitis, J Hosp Infect, J Intern Med, J Microbiol Immunol Infect, J Neurol Neurosurg Psychiatry, J Pediatr, J Virol, JAMA Ophthalmol, JCI Insight, Lancet, Lancet Gastroenterol Hepatol, Lancet Glob Health, Lancet

Healthy Longev, Lancet Infect Dis, Mayo Clin Proc, Microbiol Res, Ophthalmol Retina, PLoS Biol, PLoS Comput Biol, PLoS Med, Pediatr Pulmonol, Proc Natl Acad Sci U S A, Protein Cell, Rev Med Virol, Sci Data, Sci Total Environ, Science, Soc Sci Med, Transplantation, Vaccine, Value Health Reg Issues, Vet Res Commun, Virulence, Zoonoses Public Health

## Q2-Q4 journals of publications excluded from the systematic review

Anal Bioanal Chem, Ann Clin Lab Sci, Ann Lab Med, Ann Parasitol, Arch Razi Inst, BMC Pediatr, BMJ Mil Health, Bosn J Basic Med Sci, Cad Saude Publica, Cien Saude Colet, Clin Lab, Clin Lab Med, Clin Transplant, Diagn Microbiol Infect Dis, Disaster Med Public Health Prep, Epidemiol Prev, Eur J Med Res, Gac Sanit, Gerontology, J Assoc Physicians India, J Environ Public Health, J Formos Med Assoc, J Immunol Methods, J Infect Chemother, J Int Med Res, J Matern Fetal Neonatal Med, J Prev Med Hyg, J Theor Biol, J Virol Methods, Lab Med, Leuk Lymphoma, MSMR, Med Sci Monit, Medicina (Kaunas), Microbiol Immunol, Minerva Gastroenterol (Torino), Mol Divers, Rev Assoc Med Bras (1992), Rev Chilena Infectol, Rev Esp Salud Publica, Rev Med Inst Mex Seguro Soc, Rev Med Interne, Saudi Med J, Scand J Rheumatol, Spat Spatiotemporal Epidemiol, Transfus Med, Transfusion, Vox Sang

## Supplementary Tables

Table S1: Summary of inclusion / exclusion decisions for the systematic review.

| Characteristic | N = 640 |
|---|---|
| inclusion | |
| Duplicate | 4 (0.6%) |
| Excluded: conflict of interest | 5 (0.8%) |
| Excluded: foreign language | 7 (1.1%) |
| Excluded: not Covid-19 | 2 (0.3%) |
| Excluded: not in humans | 9 (1.4%) |
| Excluded: secondary research | 22 (3.4%) |
| Excluded: did not assess seroprevalence | 233 (36%) |
| Excluded: risk factors or subgroups | 41 (6.4%) |
| Excluded: no full text | 3 (0.5%) |
| Sensitivity analysis: research letter | 23 (3.6%) |
| Included | 291 (45%) |

[1] n (%)

Table S2: Proportion of studies 1) using any correction for test sensitivity and specificity and 2) using either Rogan-Gladen or Bayesian approaches to correction, by start of sampling period and type of journal Confidence intervals have been computed using the Clopper-Pearson method and are only reported if the total sample size was greater than 5.

| start of sampling | any correction | Rogan-Gladen | Bayesian |
|---|---|---|---|
| **publications in high impact journals** | | | |
| 2019-Q4 | 2/5 (40.0%) | 1/2 (50.0%) | 1/2 (50.0%) |
| 2020-Q1 | 4/10 (40.0%; 12.2 to 73.8) | 1/4 (25.0%) | 2/4 (50.0%) |
| 2020-Q2 | 12/53 (22.6%; 12.3 to 36.2) | 5/12 (41.7%; 15.2 to 72.3) | 5/12 (41.7%; 15.2 to 72.3) |
| 2020-Q3 | 10/34 (29.4%; 15.1 to 47.5) | 6/10 (60.0%; 26.2 to 87.8) | 4/10 (40.0%; 12.2 to 73.8) |
| 2020-Q4 | 14/44 (31.8%; 18.6 to 47.6) | 8/14 (57.1%; 28.9 to 82.3) | 6/14 (42.9%; 17.7 to 71.1) |
| 2021-Q1 | 11/33 (33.3%; 18.0 to 51.8) | 7/11 (63.6%; 30.8 to 89.1) | 3/11 (27.3%; 6.0 to 61.0) |
| 2021-Q2 | 4/18 (22.2%; 6.4 to 47.6) | 3/4 (75.0%) | 1/4 (25.0%) |
| 2021-Q3 | 3/9 (33.3%; 7.5 to 70.1) | 2/3 (66.7%) | 1/3 (33.3%) |
| 2021-Q4 | 0/6 ( 0.0%; 0.0 to 45.9) | – | – |
| 2022-Q1 | 0/3 ( 0.0%) | – | – |
| 2022-Q2 | 0/1 ( 0.0%) | – | – |
| **publications in other journals** | | | |
| 2019-Q4 | 1/2 ( 50.0%) | 1/1 (100.0%) | 0/1 ( 0.0%) |
| 2020-Q1 | 0/6 ( 0.0%; 0.0 to 45.9) | – | – |
| 2020-Q2 | 4/28 ( 14.3%; 4.0 to 32.7) | 2/4 ( 50.0%) | 0/4 ( 0.0%) |
| 2020-Q3 | 4/23 ( 17.4%; 5.0 to 38.8) | 4/4 (100.0%) | 0/4 ( 0.0%) |
| 2020-Q4 | 2/18 ( 11.1%; 1.4 to 34.7) | 1/2 ( 50.0%) | 0/2 ( 0.0%) |
| 2021-Q1 | 1/9 ( 11.1%; 0.3 to 48.2) | 0/1 ( 0.0%) | 0/1 ( 0.0%) |
| 2021-Q2 | 0/2 ( 0.0%) | – | – |
| 2021-Q3 | 0/7 ( 0.0%; 0.0 to 41.0) | – | – |
| 2021-Q4 | 0/1 ( 0.0%) | – | – |
| 2022-Q1 | 0/1 ( 0.0%) | – | – |
| 2022-Q3 | 0/1 ( 0.0%) | – | – |

Table S3: Changes in expected bias or bias category over time, by type of journal.

| Group | Characteristic | 2020-Q1 | 2020-Q2 | 2020-Q3 | 2020-Q4 | 2021-Q1 | 2021-Q2 | 2021-Q3 |
|---|---|---|---|---|---|---|---|---|
| high impact | number of studies | N = 2 | N = 22 | N = 13 | N = 18 | N = 12 | N = 7 | N = 3 |
| | expected bias | | | | | | | |
| | Median | 1.8 | 0.3 | 0.0 | 0.0 | 0.0 | -2.1 | 0.1 |
| | Min - Max | 0.0 - 3.6 | -1.9 - 4.0 | -11.8 - 4.0 | -2.5 - 4.5 | -4.3 - 1.6 | -12.2 - 1.0 | -0.3 - 0.3 |
| | bias category | | | | | | | |
| | [-15,-10) | 0 (0%) | 0 (0%) | 1 (8%) | 0 (0%) | 0 (0%) | 2 (29%) | 0 (0%) |
| | [-10,-5) | 0 (0%) | 0 (0%) | 1 (8%) | 0 (0%) | 0 (0%) | 1 (14%) | 0 (0%) |
| | [-5,-1) | 0 (0%) | 1 (5%) | 0 (0%) | 5 (28%) | 4 (33%) | 1 (14%) | 0 (0%) |
| | [-1,1) | 1 (50%) | 16 (73%) | 9 (69%) | 8 (44%) | 6 (50%) | 2 (29%) | 3 (100%) |
| | [1,5) | 1 (50%) | 5 (23%) | 2 (15%) | 5 (28%) | 2 (17%) | 1 (14%) | 0 (0%) |
| | [5,10] | 0 (0%) | 0 (0%) | 0 (0%) | 0 (0%) | 0 (0%) | 0 (0%) | 0 (0%) |
| other | number of studies | N = 3 | N = 12 | N = 5 | N = 10 | N = 4 | N = 2 | N = 3 |
| | expected bias | | | | | | | |
| | Median | 0.1 | 0.3 | -0.1 | 0.0 | -0.1 | -0.3 | -4.1 |
| | Min - Max | -0.3 - 0.7 | -0.5 - 7.1 | -6.3 - 3.7 | -4.3 - 5.2 | -1.5 - 0.2 | -0.5 - -0.1 | -5.0 - 0.6 |
| | bias category | | | | | | | |
| | [-15,-10) | 0 (0%) | 0 (0%) | 0 (0%) | 0 (0%) | 0 (0%) | 0 (0%) | 0 (0%) |
| | [-10,-5) | 0 (0%) | 0 (0%) | 1 (20%) | 0 (0%) | 0 (0%) | 0 (0%) | 1 (33%) |
| | [-5,-1) | 0 (0%) | 0 (0%) | 1 (20%) | 3 (30%) | 1 (25%) | 0 (0%) | 1 (33%) |
| | [-1,1) | 3 (100%) | 10 (83%) | 2 (40%) | 4 (40%) | 3 (75%) | 2 (100%) | 1 (33%) |
| | [1,5) | 0 (0%) | 1 (8%) | 1 (20%) | 2 (20%) | 0 (0%) | 0 (0%) | 0 (0%) |
| | [5,10] | 0 (0%) | 1 (8%) | 0 (0%) | 1 (10%) | 0 (0%) | 0 (0%) | 0 (0%) |

[1] N = N; n (%)

Table S4: Key outcomes of systematic review. The sensitivity analysis included 291 publications meeting all inclusion criteria, but not any research letters meeting all other inclusion criteria.

| Characteristic | Overall<br>N = 291 | high impact<br>N = 194 | other journals<br>N = 97 |
|---|---|---|---|
| start_sampling | | | |
| Median | 2020-09-21 | 2020-10-15 | 2020-09-15 |
| (Min - Max) | (2019-10-15 - 2022-04-26) | (2019-10-15 - 2022-04-26) | (2019-11-15 - 2022-01-20) |
| method_report | | | |
| corrected and fully reported Se/Sp | 64 (22%) | 53 (27%) | 11 (11%) |
| corrected and no/partial information | 3 (1.0%) | 2 (1.0%) | 1 (1.0%) |
| uncorrected and fully reported Se/Sp | 120 (41%) | 80 (41%) | 40 (41%) |
| uncorrected and no/partial information | 104 (36%) | 59 (30%) | 45 (46%) |
| methods | | | |
| Bayesian | 19 (28%) | 19 (35%) | 0 (0%) |
| Rogan-Gladen | 40 (60%) | 32 (58%) | 8 (67%) |
| unspecified method | 8 (12%) | 4 (7.3%) | 4 (33%) |
| Se | 95.2 (60.2 - 100.0) | 95.0 (60.2 - 100.0) | 96.8 (72.2 - 100.0) |
| Sp | 99.6 (82.4 - 100.0) | 99.6 (82.4 - 100.0) | 99.8 (92.5 - 100.0) |
| bias | 0.00 (-12.20 - 9.10) | -0.03 (-12.20 - 9.10) | 0.09 (-6.30 - 7.15) |
| bias_cat | | | |
| [-15,-10) | 5 (2.7%) | 5 (3.8%) | 0 (0%) |
| [-10,-5) | 6 (3.3%) | 4 (3.0%) | 2 (3.9%) |
| [-5,-1) | 29 (16%) | 20 (15%) | 9 (18%) |
| [-1,1) | 113 (61%) | 81 (61%) | 32 (63%) |
| [1,5) | 28 (15%) | 22 (17%) | 6 (12%) |
| [5,10] | 3 (1.6%) | 1 (0.8%) | 2 (3.9%) |

[1] n (%); Median (Min - Max)
